# Exploration of a virtual restoration practice route for architectural heritage based on evidence-based design: a case study of the Bagong House

**DOI:** 10.1186/s40494-023-00878-8

**Published:** 2023-02-20

**Authors:** Ziyi Zhang, Yiquan Zou, Wei Xiao

**Affiliations:** 1 Hubei University of Technology, Wuhan, Hubei Province China; 2 CITIC Architectural Design and Research Institute Co., Wuhan, China

**Keywords:** Virtual restoration, Architectural Heritage Preservation, Evidence-based design, Evidence-based medicine, Digital restoration

## Abstract

Architectural heritage is a testament to human and natural development, and the process of human social development can be glimpsed through the study and exploration of heritage. However, in the long history of human social development, architectural heritage is vanishing, and protecting and restoring such heritage is a pressing issue in contemporary society. This study applies the evidence-based theory of medicine to the virtual restoration practice of architectural heritage, which focuses more on scientific data-driven research and decision-making than does traditional restoration. Combined with the practice of evidence-based medicine, the stages of digital conservation of architectural heritage for virtual restoration based on evidence-based design are investigated, forming a comprehensive knowledge system consisting of clear objectives, evidence-based research, evidence assessment, virtual restoration-guided practice, and post feedback. In addition, it is emphasized that the restoration of architectural heritage should be founded on the outcomes obtained through evidence-based practice that have been translated into evidence, in turn creating a rigorous evidence-based system with high-frequency feedback. The final illustration of the procedure is the Bagong House in Wuhan, Hubei Province, China. The examination of this practice line provides a scientific, humanistic, and practicable theoretical framework for the restoration of architectural heritage and fresh ideas for the restoration of other cultural assets, which have significant practical application value.

## Introduction

The 50th anniversary of the signing of the Convention Concerning the Protection of the World Cultural and Natural Heritage is being observed in 2022. The World Heritage System has a special allure because it connects the shared sentiments of humanity and documents the evolution of human civilization. Nevertheless, the world in which we live is facing problems that have never been seen in the twenty-first century. The extinction of heritage is being sped up by things like the new coronavirus pandemic and the destruction of the environment [1]. As a result of the rapid development of digital and internet technologies, which have significantly altered the face of architectural heritage conservation, we must study and practice extensively how to apply the most cutting-edge digital technology to the field of architectural heritage conservation.

Evidence-based design is a methodology that employs the concept of evidence-based medicine in the engineering industry. Such design aims to move away from the traditional design approach based on specifications, which relies on the personal experience and assumptions of the supervisor, and toward a design model that focuses on the completion of the effect and actual use and attaches importance to the accumulation and recycling of evidence to guarantee that design decisions are justifiable and traceable and to develop a more scientific and rigorous work design model [2]. In 2000, the American Center for Health Design, in collaboration with several medical organizations and architects, launched the Pebble Project, a collaborative research program that uses evidence-based design methods to conduct research on medical buildings and their architectural design, with the goal of amassing additional reference data for medical design organizations and their clients. The initiative intends to collect more reference data for healthcare design organizations and their clients and to create an evidence-based design database. More than forty hospitals and care facilities have joined the effort, with the number of participating organizations continuing to grow [2–4].

In 2008, the Center for Architectural Heritage Preservation at Texas A&M University in the United States assembled a multidisciplinary team of architects, engineers, geologists, historians, and students to collect evidence-based data for the conservation and enhancement of the A-listed French site Pointe d'Oc on the Normandy coast. This situation is a classic instance of evidence-based design, which emphasizes the identification of core problems in practice through research, the rational and rigorous search for the best evidence-based decisions, the high degree of integration between scientific research and practical application, and the significance of evaluation and feedback on the results.

The virtual restoration of architectural heritage not only is a secondary way to keep history alive but also goes beyond time and space, acting as both a conversation with the past and a point of reference for the present [5].

In 1995, the United Kingdom held a conference on "Virtual World Heritage" and presented the virtual restoration of Stonehenge, which marked the beginning of the virtual restoration of architectural heritage. Among the international cases of architectural heritage restoration, the most renowned is the world's largest virtual architectural heritage system, which took ten years to construct, in Italy's ancient capital city [6], Rome. The Digital Dunhuang project team was formed in the 1990s by more than a dozen architectural conservancies, including the Dunhuang Academy of Art in China, the Oriental Institute in the United Kingdom and Russia, and the Mellon Foundation in the United States, to conduct the virtual restoration of Dunhuang art [7]. In 2003, professionals and intellectuals from China and Japan labored for 22 months to complete the virtual restoration of the "Digital Forbidden City" [8]. Anna Osello et al. argued that the growth of heritage building information modeling (HBIM) has created new potential for digital media to penetrate the global heritage conservation profession [9]. Using HBIM and virtual restoration techniques to optimize and accelerate the emergency restoration of a historic castle in Piedmont, Soto-Martin et al. [10] used virtual reality and other techniques to reconstruct the architectural structure and frescoes of the Church of St. Augustine in La Laguna over the past two years.

The two categories of virtual restoration are exterior decorative restoration and structural restoration. In the case of structural restoration, when it is difficult to determine whether physical restoration will damage the structure, virtual restoration is performed in a computer environment beforehand, the force analysis of the structure to be restored is evaluated, and physical restoration is performed only after it has been determined that the restored structure is reasonable, thereby preventing secondary damage caused by humans. The emphasis on virtual repair does not imply the abandonment of actual restoration. The digital information of architectural heritage has the same precision as that of physical artifacts and can complement appropriate theoretical investigations with evidence. Virtual repair in a digital system is reversible, but actual entities cannot be restored once damaged.

**Fig. 1:**
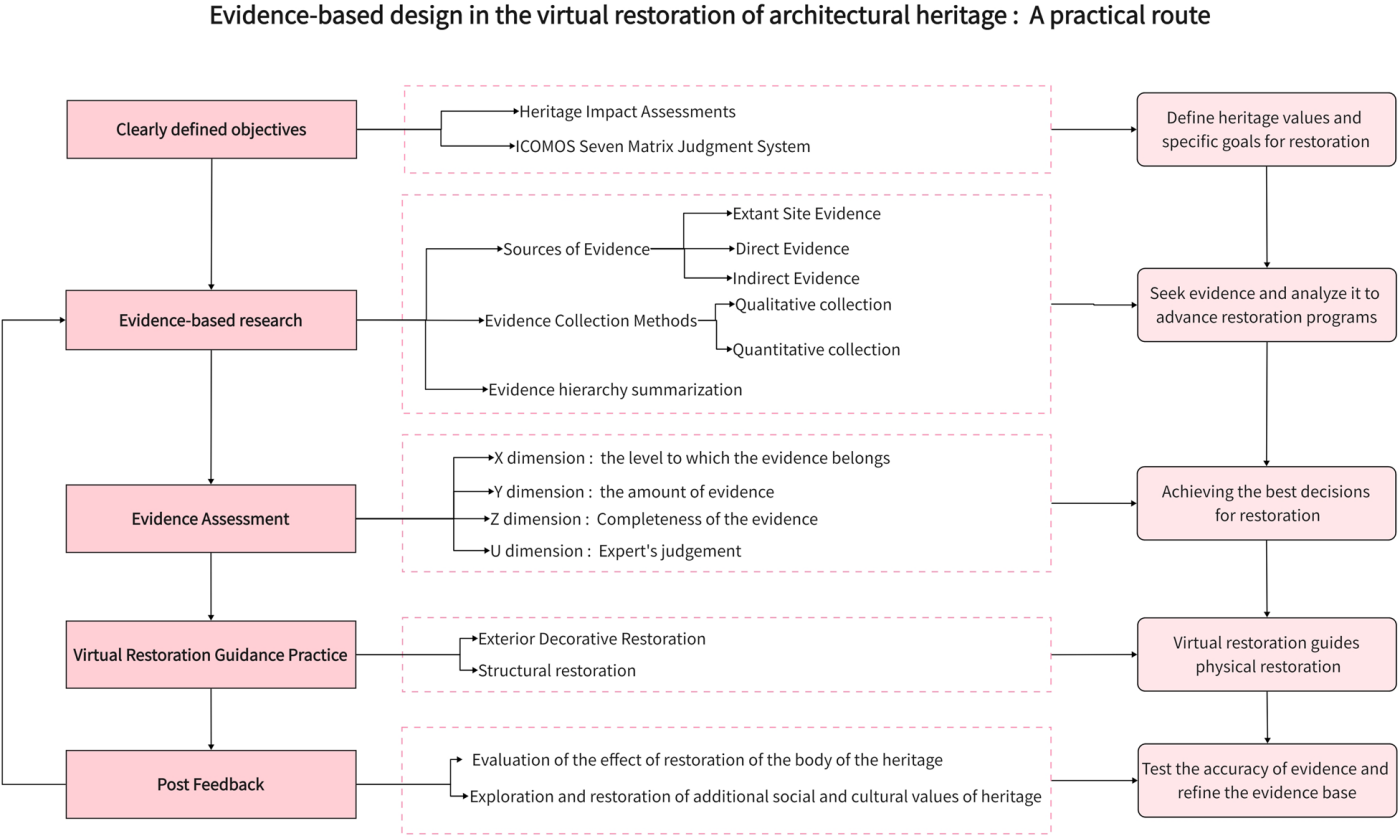
The framework of the article

This article first introduces the development history of virtual restoration and the background of evidence-based design. After that, inspired by the theory and practice of evidence-based medicine, it explores the stages of realization of virtual restoration of architectural heritage digital conservation based on evidence-based design, which provides a strong theoretical research basis for digital conservation and restoration of architectural heritage. A practical route of evidence-based design in virtual restoration of architectural heritage is proposed in the methods and materials section: clearly defined objectives—evidence-based research—evidence assessment—virtual restoration guidance practice—post-feedback, as the theoretical basis of this paper. Finally, the application process is illustrated with the example of the Bagong House in Wuhan, Hubei Province, China. The framework of the article is shown in the Fig. 1.

## Methods and materials

### Related background

#### Semantic evolution of virtual restoration

**Fig. 2:**
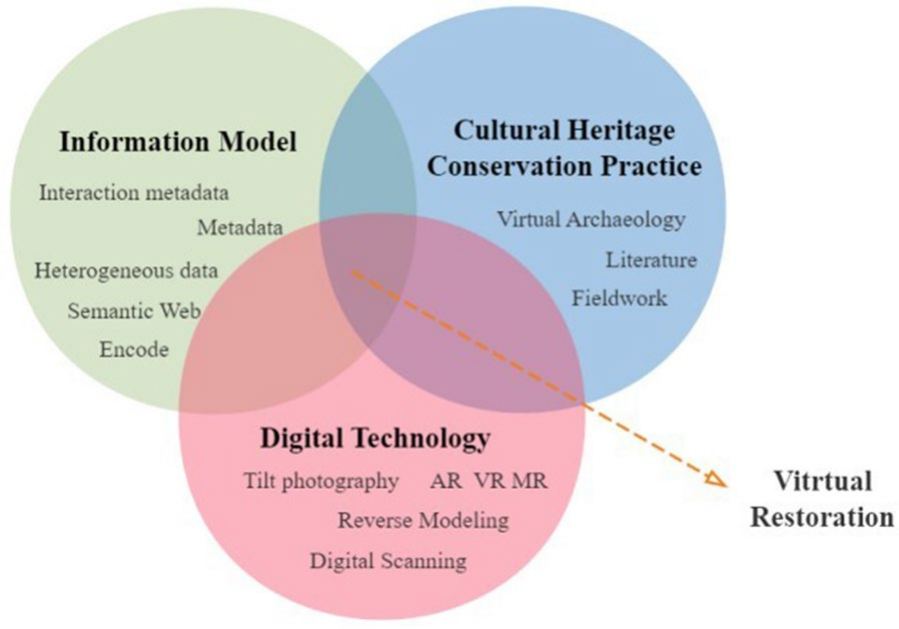
Semantic relations for virtual restoration

Virtual restoration, also known as simulation restoration or digital restoration, is based on the multilevel information of architectural heritage (such as images, point clouds, and documents), combined with traditional architectural conservation science and technology, and employs modern technologies such as computer graphics, image processing, web space, and virtual information to achieve the automatic restoration of building geometry, texture, and structure. The diagram in Fig. 2 illustrates the semantic link.

**Fig. 3:**
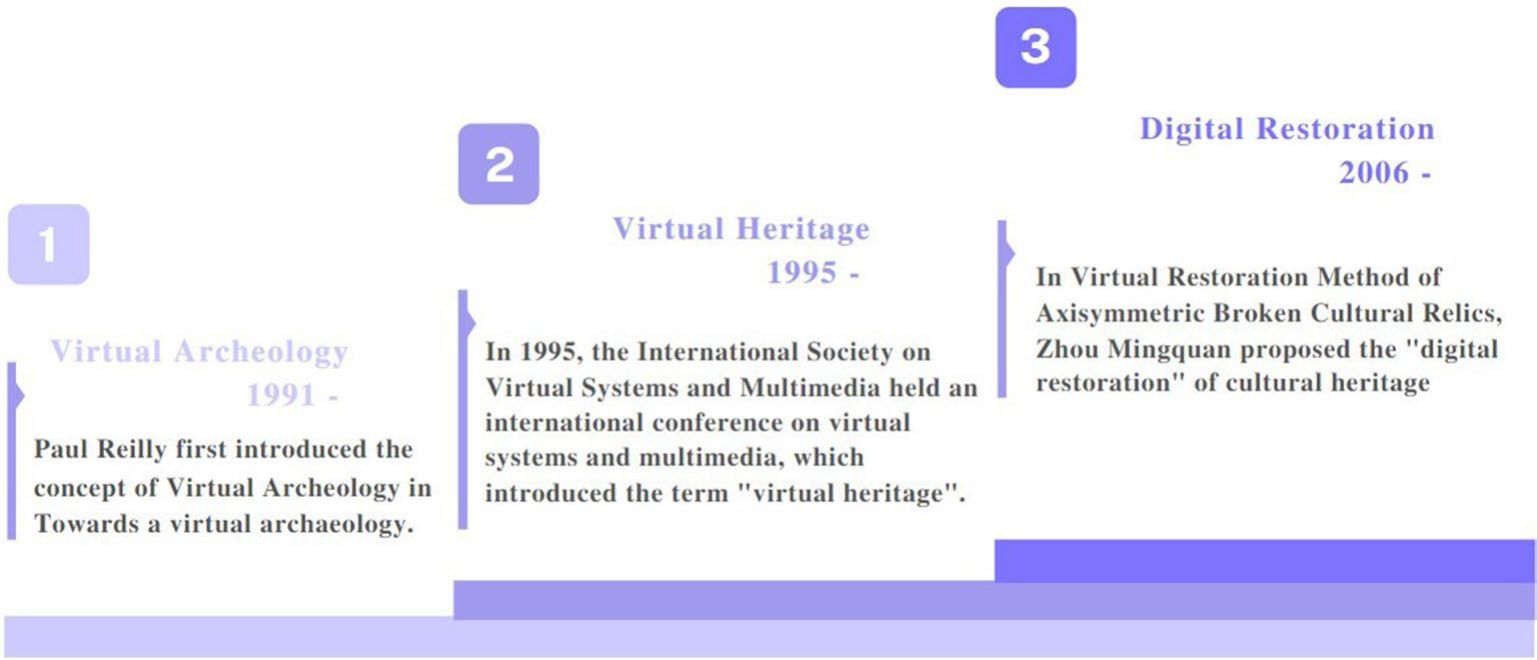
Evolution of virtual restoration

The virtual restoration of architectural heritage is a comprehensive applied technical science involving interdisciplinary and multidisciplinary synergy, with technical disciplines including chemistry, physics, environmental protection, architecture, mineralogy and petrology, computer information technology, etc., and closely related to social disciplines including museology, library science, archives, archaeology, etc. Its evolution has involved three stages: virtual archaeology, virtual heritage, and digital restoration. For more information, see Fig. 3.

##### Virtual archaeology

The creation of virtual restoration stemmed from the foundation of archaeology, and researchers had practiced virtual archaeology and virtual heritage prior to the formation of the idea. Wilcock et al. [11] used computer technology for the virtual reconstruction of architectural heritage sites in 1973, Arnold et al. [12] used computer technology for archaeology research in 1989, and Reilly P [13]. used IBM's British Science Center's WINSOM's Winchester SOLID modeling system to virtually reconstruct a model of Winchester Cathedral in England in the same year.

Paul Reilly did not propose the concept of "virtual archaeology" for the first time until 1991 in “Towards a Virtual Archaeology” [14], suggesting that "when a building or structure has disappeared or is in a poor state of preservation, it should be studied using computer technology in the form of three-dimensional visualization and virtual presentation of reality."

In 1998, Computer Applications and Quantitative Methods in Archaeology (CAA) hosted a conference on virtual reality in archaeology and published “Virtual Reality in Archaeology: Computer Applications and Quantitative Methods in Archaeology”, which explains the concept of virtual reality in archaeology in detail. In the 21 century, virtual archaeology has continued to develop into a distinct field [15].

##### Virtual legacy

**Fig. 4:**
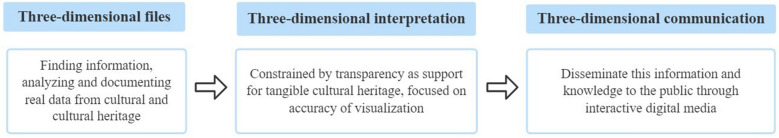
Virtual heritage content

In 1995, the International Society on Virtual Systems and Multimedia conducted an international conference on virtual systems and multimedia and formally proposed the phrase "virtual heritage" to characterize architectural heritage and virtual reality, i.e., the digitalization of architectural history [16, 17]. At the meeting, the phrase "virtual heritage" was formally introduced. As a component of digital heritage, "virtual heritage" is the virtual reproduction of architectural heritage in a computer environment and its representation via a digital interface, allowing for a degree of immersion and interactivity [18]. Virtual heritage is an example of the application of virtual heritage as a component of digital heritage, which is a virtual reproduction of architectural heritage in a computer environment visualized through a digital interface that provides a degree of immersion and interaction and reconstructs a navigable three-dimensional world. Virtual heritage entails, in a way, synthesis, conservation, replication, reproduction, digital in-processing, and presentation utilizing cutting-edge image technology [19]. Including three specific phases [20]: 3D documentation, 3D interpretation and 3D communication, as shown in the Fig. 4.

##### Digital restoration

The digital restoration of architectural heritage began in 2006 with the release of an article that placed greater emphasis than that in the previous two phases on the automation of architectural heritage restoration [21, 22]. The degree of automation in the restoration of architectural heritage was highlighted in the first two phases of digital restoration. The academic definitions of "digital restoration" and "virtual restoration" had not been clearly differentiated when the semantics of virtual restoration progressed to the point of "digital restoration." There is no clear distinction between "digital restoration" and "virtual restoration," as both terms refer to the use of computer graphics, image processing, and virtual information technologies to automate the restoration of building geometry and texture based on high-precision three-dimensional models in conjunction with traditional architectural conservation and restoration work [23].

The introduction of digital restoration technology into the field of heritage conservation has become a common aim among scholars from various countries, whether in paper-cut patterns [24], clothing [25], other movable areas, the Great Wall [26], the Terracotta Warriors and Horses [27], frescos [28, 29], wooden churches [30], ancient sites [31], and watchtowers [32]. Therefore, the application of digital restoration to architectural heritage is the topic of this article.

##### Evidence-based medicine

**Fig. 5:**
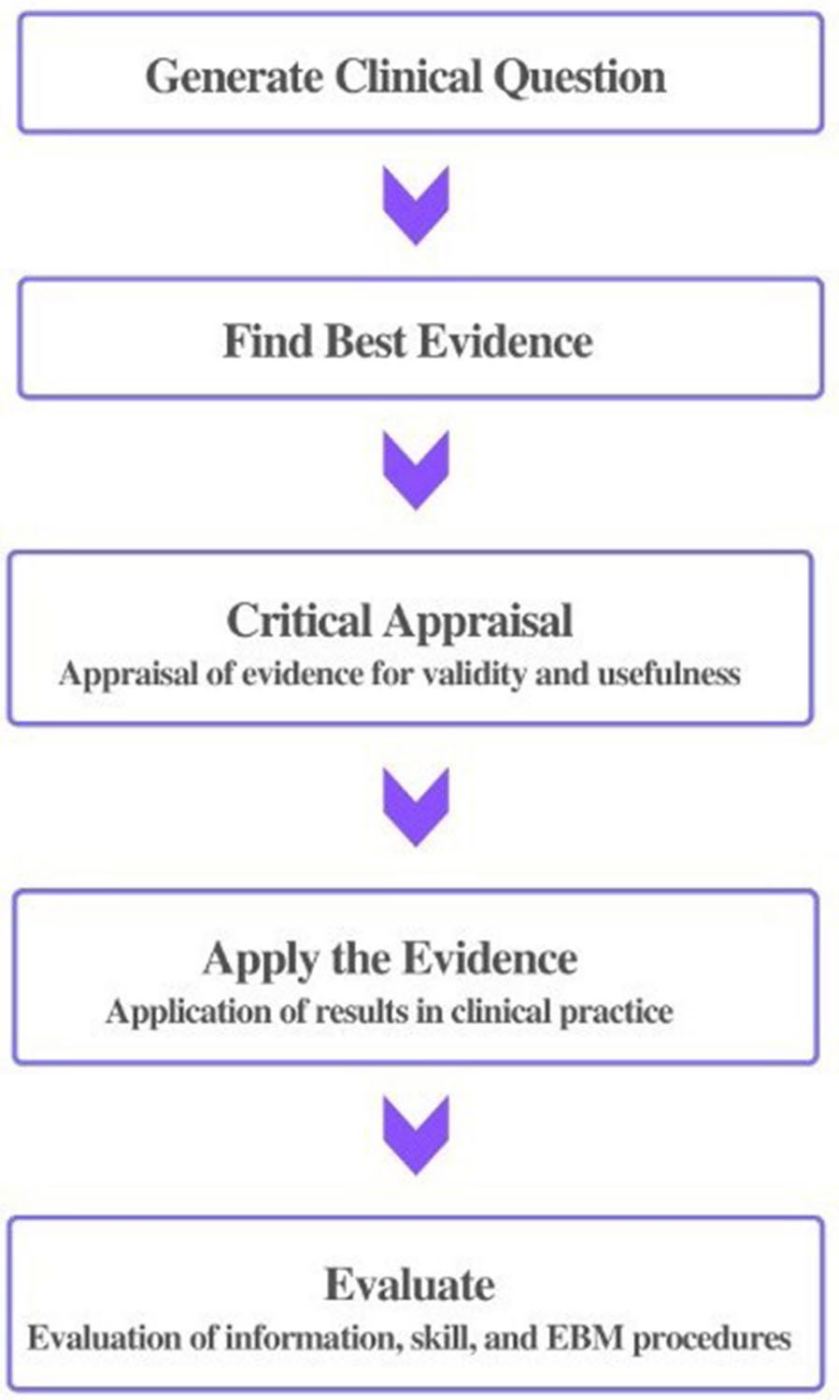
Process of evidence-based medicine

Evidence-based medicine is the process of systematically discovering, evaluating, and applying well-designed results from well-designed and executed clinical studies to optimize and implement clinical decisions [33]. Academically, evidence-based medicine is defined as "the integration of the best available research evidence, clinical expertise, and patient values to determine the care of individual patients" [34, 35]. The following Fig. 5 are the steps of evidence-based medicine practice, which is comparable to the practice of evidence-based design in the virtual restoration of architectural heritage.

##### Evidence-based design

Evidence-based medicine, which promotes that the practice of medicine should be based on the rigor of research methodologies and the dependability of research outcomes, is where the philosophy of evidence-based design (EBD) first emerged [36]. In 1984, Ulrich used the first scientific evidence-based methodology to validate the impact of natural landscapes on patient recovery [37], looking at the association between postsurgical patient recovery and window views in hospitals [38]. The above article was the first to confirm the benefit of natural settings for patient recovery using scientific evidence. When compared to conventional design techniques, evidence-based design places more emphasis on research and decision-making based on scientific data, giving design choices a rationale supported by scientific methodology [39]. The design process is changed into a "research-design-research" cycle, the results of which are more rigorous and scientific because feedback and experience are applied to succeeding designs. D. Kirk Hamilton introduced the term in 2004.

**Fig. 6:**
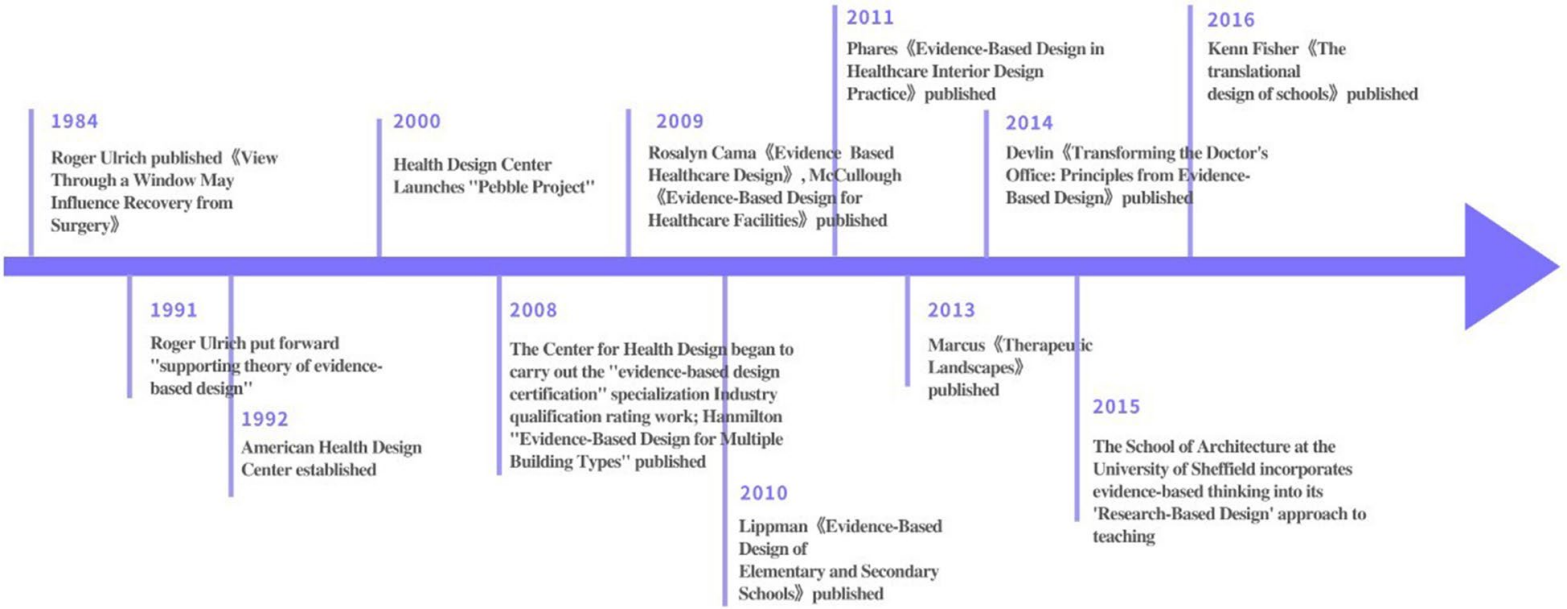
Evolution of evidence-based design

"Evidence-based design" is defined as "a process in which architects collaborate with their customers to carefully and purposefully use and analyze the most trustworthy scientific evidence available to make educated decisions about design-related decisions." "Sound decisions about design-related issues result from this." In the field of medical architecture, where it has become the standard research methodology in both Europe and the United States, evidence-based design was first applied. The relationship between evidence-based design and historic building conservation was defined by Carolyn Peterson as follows in 2007, when Europe, the United States, and other developed nations formally applied evidence-based design to the conservation of historic buildings: "Historic design is the most suitable discipline in the field of architecture for the preservation, restoration, and adaptation of structures and environments." "An evidence-based design approach, which incorporates the full preservation process and directs all decision-making processes from the commencement of a project to its final details, is better suited for the preservation, restoration, and adaptive adaptation of structures and surroundings."[40] Figure 6 depicts the evolution of evidence-based design research over time.

A damaged building is virtually restored on a computer, which then directs the actual restoration, with each step backed up by solid scientific data. Heritage is frequently viewed as an organism with "vital functions" from an ontological perspective. The "diagnosis and restoration" of its "organic" functions is conceptually and methodologically similar to the treatment of living organisms or cases, and the logic of the virtual restoration of architectural heritage has a framework that is similar to evidence-based design [41]. Like evidence-based design, the logic of the virtual restoration of architectural heritage follows a similar path.

#### Closed-loop theory

**Fig. 7:**
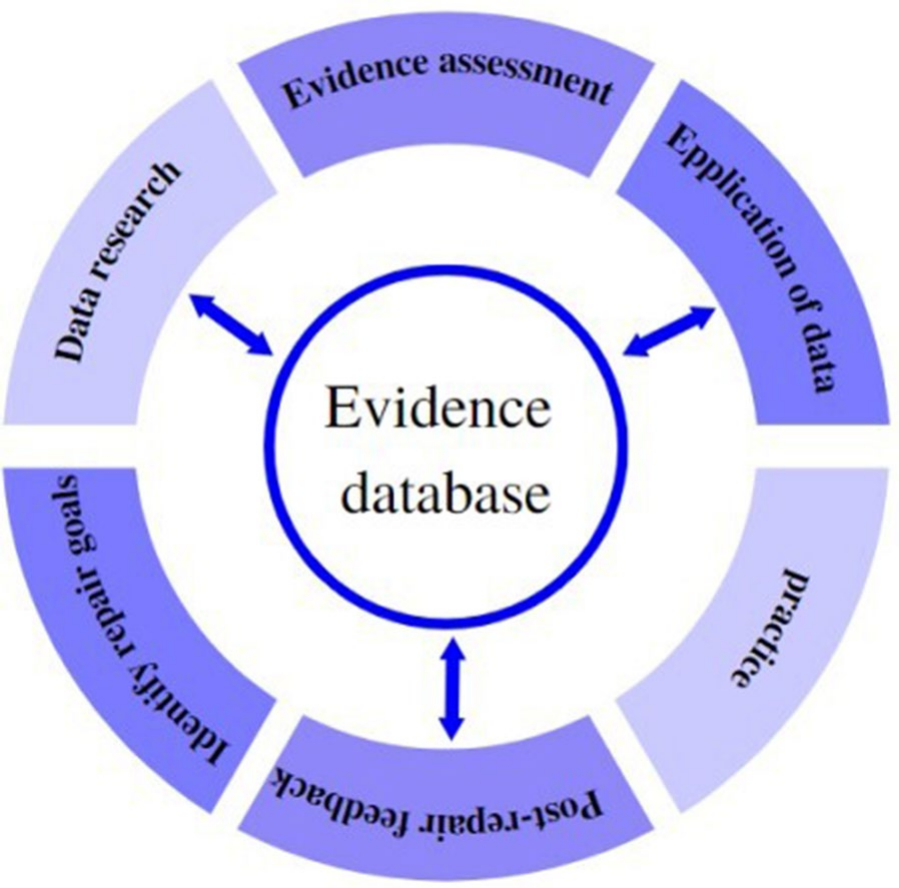
Closed-loop theory

The “closed-loop” control theory concept is crucial because the digital preservation of architectural history is a dynamic, ongoing developmental process. The workflow from proposal development, evidence search, virtual restoration, expert assessment of feasibility, and post restoration feedback forms a dynamic closed-loop structure in the evidence-based design practice of the virtual restoration of architectural heritage, which is in line with the core principles of heritage conservation. The concept of "feedback" is crucial to the closed-loop structure, in which some system components control themselves and monitor their objectives through negative feedback to reach a stable, balanced, and sustainable state [42]. As indicated in the Fig. 7, during the virtual and physical restoration phases of architectural heritage, a closed process of operational optimization and knowledge construction is created by transforming the evidence-based practice results into evidence.

The creation and use of evidence procedures alternate repeatedly, and the end of one round of conservation signals the start of a new round of conservation in the ideal state of digital conservation of architectural heritage. This process is continuous and closed loop, with a post feedback mechanism that sets off a negative feedback mechanism to keep the digital conservation process stable and moving forward [41].

### Evidence-based design in the virtual restoration of architectural heritage: a practical route

#### Clearly defined objectives

The International Council on Monuments and Sites (ICOMOS) and the United Nations Educational, Scientific, and Cultural Organization (UNESCO) collaborated to create the "Guidelines on Heritage Impact Assessments for Cultural World Heritage Properties" in 2009, which formally introduced heritage impact assessments (HIAs) as a management technique in the field of heritage conservation [43]. HIAs were formally introduced as a management strategy for heritage conservation in 2011 by the Guidelines on Heritage Impact Assessments for Cultural World Heritage Properties [44]. HIAs are increasingly being used internationally in Commonwealth nations like the UK, Canada, Australia, and South Africa. These assessments are derived from the Environmental Impact Assessment (EIA), a sustainable development-based approach to historic conservation management. The majority of Commonwealth nations, including the UK, Canada, Australia, and South Africa, have implemented HIAs in the field of historic conservation.

As a technical approach and managerial tool, HIAs are anticipatory and forward looking. To slow down the rate of disintegration of heritage and its surroundings and to maintain and manage heritage scientifically, many methods of improvement, restoration, and human management are implemented. By using HIAs at the initialization stage of virtual restoration, the legacy is valued, and restoration objectives are suggested as a result. The restoration team should propose restoration goals by fusing various areas of expertise, and based on these goals, key research questions that may arise in the restoration process and elicit numerous research subquestions that will serve as the foundation for the second stage of obtaining evidence to support the questions should be refined.

**Fig. 8:**
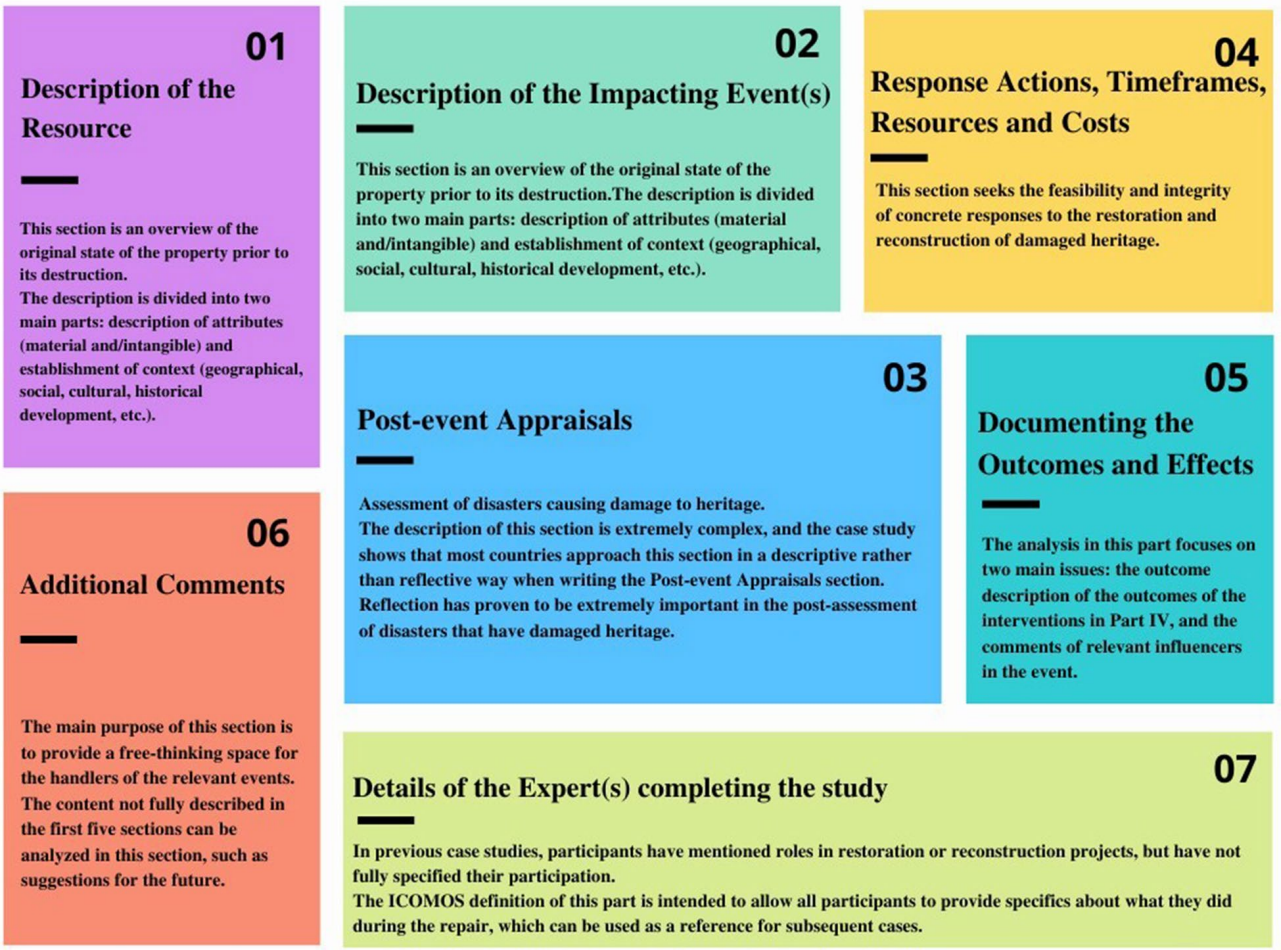
Seventh ICOMOS matrix judging system

To explore a set of guiding lessons with universal applicability, ICOMOS conducted a study and analysis of 11 case studies of the restoration and reconstruction of architectural heritage in 2019. The above authors did this in collaboration with the International Centre for the Study of the Preservation and Restoration of Cultural Property and released the "ICOMOS Seven Matrix Judging System" [45], as shown in the Fig. 8. The system is not a hard and necessary prescription but rather a guideline for compatibility, and it should be noted that this system can be used as a standard for post restoration assessment and a reference for prerestoration planning. The system can be used as a guide for virtual restoration preprogramming, with descriptions of resources, response actions, time frames, resources and costs, outcomes and impacts, further comments, and information on those experts who completed the study.

#### Evidence-based research

The next step, according to evidence-based design theory, is to find and analyze evidence to advance the protocols after the visualization of the restoration goals and protocols has been developed. The content of the evidence-based research phase can be divided into two categories: multisource data collection and data hierarchical induction.

Finding trustworthy sources of data to produce an original proof is important prior to data collection. There are generally three main categories due to the variety of information sources and content involved. The information extracted from the conclusions of previous research results and verified sources, such as books, academic literature, project documentation, old photos or photocopies, that provide descriptive or documentary evidence of the target falls into two categories: the first is evidence information about the current state of existing sites, which is extracted digitally, and the second is information extracted from these sources. The third sort of evidence, which is indirect and completes the first two types, is the analysis of related cases [46].

Quantitative and qualitative data collection techniques are the two basic categories. Quantitative research seeks to further refine people's understanding of the subject of study to reveal the law more precisely, whereas qualitative research uses induction and deduction, analysis and synthesis, abstraction and generalization, etc., to process the data obtained for the processing of thought^2,49^. To expose the rules more scientifically, quantitative research aims to increase people's comprehension of the research object.

Wu et al. [47] classified data into "structured data" and "unstructured data" according to their type. The former is composed of information that can be used with the Internet of Things (IoT), 3D modeling, geographic information systems (GIS), multisource remote sensing (RS), and satellite positioning systems (GPS), referring to structured data that were obtained through the quantitative analysis of various scales and dimensions, covering spatial positioning, geometric forms, and attributes, using spatial information technologies such as GPS, geographic information systems, multisource remote sensing (RS), the Internet of Things, 3D simulation, photogrammetry, drone images, and 3D laser point clouds [26, 48, 49]. The latter consists of unstructured data gathered through qualitative examination of archival knowledge, building methods, historical texts, and textual documentation of the endangered architectural legacy. Data gathering is done in the following ways throughout the phase of evidence-based research on the virtual restoration of architectural heritage [2, 39, 50]:

**Literature research**: The current body of literature is methodically compiled and examined for identification.

**Document collection and analysis**: Contrary to the "external information" of extant research, document collection and analysis take place primarily in internal information systems, like libraries and design institutes, and consist primarily of plans, drawings, construction technical documents, maintenance records, and changes to the project under study.

**Site exploration and measurement**: In contrast to the "external information" of extant research, document collection and analysis mostly occur in internal information systems like libraries and design institutes. Plans, drawings, construction technical documents, maintenance records, and changes to the project being studied are the most common types of documents collected and analyzed.

**Questionnaire**: A mixed questionnaire with both closed- and open-ended questions can be used to collect data for evidence-based research. After the questionnaire is compiled, it is given out in person, over the phone, by email, by mail, or in interviews.

**On-site interviews**: The questionnaire's disadvantage is that it is entirely written and cannot be modified or expanded randomly. To acquire a more comprehensive and in-depth understanding of the data, they must be combined with on-site interviews.

**Behavioral observation:** By observing the pertinent phenomena at architectural heritage sites and the behavior patterns of the pertinent users, it is a good complement to questionnaires and on-site interviews to gather useful information. Table 1 below breaks down behavioral observations into two categories: unstructured and structured data.

**Table 1:** Behavioral observation categories

	Classification	Meaning
Unstructured	Casual observation	Quick visual inspection without predefined categories
	Participant observation	The observer is involved, playing or being part of a user and the environment being observed
	Trace observation	Observe or look for physical traces of evidence Trace observation is further divided into loss traces and cumulative traces
Structured	Systematic observation	Predesign a score sheet to record data
	Behavior map	Create a map or drawing similar to a floor plan, using various behavioral symbols, to record the user's behavior patterns

Table 2 shows the data sources and how they are collected during the multisource data collection phase.

**Table 2:** Multisource data collection

Data Category	Data Content	Source	Collection Methods
Structured data	Status quo geography	Obtained using Global Positioning System (GPS), geographic information system (GIS), photogrammetry, drone imagery, etc	Document collection and analysis Field exploration and measurement
Unstructured data	Field literature	Academic websites, academic monographs, thematic databases, etc	Document collection and analysis Literature research questionnaire On-site interviews Behavioral observations
	Expert commentary	Academic forums, fund organizations, academic websites, academic lectures, etc	
	Folk tale	Word of mouth of the original inhabitants, etc	

**Fig. 9:**
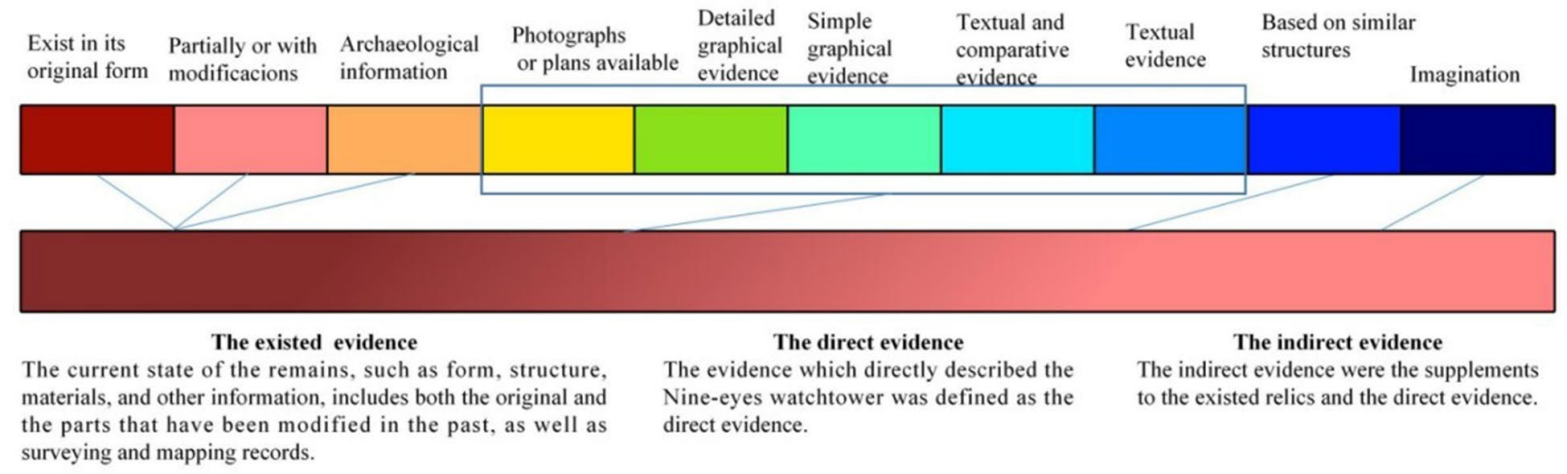
Classification of evidence (Image source [56])

By fusing the traits of various data sources with the precise target identification of the restoration program, various types of data should be extracted as evidence for data hierarchy summarization. Portus Theodosiacus introduced the "concept of multiscale evidence" in 2018 [51] and used it to inform the Byzantine 1200 restoration project. However, this high-saturation, non-gradient color classification method does not visually reflect the gradient change in evidence validity. Moreover, Liu et al. [52], in 2020, proposed establishing three levels of evidence hierarchy, transforming different attribute evidence to evidence hierarchy based on the strength and weakness of the evidence, and improving the evidence evaluation mechanism of decontextualization. Combining the research ideas of the above two groups of scholars, Li Z et al. regrouped the scale of evidence and established three levels of gradient patterns of 10 categories of homochromatic evidence hierarchy, and the scale of evidence can be summarized as extant relic, direct and indirect evidence [26, 53], as shown in the Fig. 9. This study adopts mainly this hierarchical classification.

#### Evidence assessment

Data-based decision-making, or making restoration decisions based on solid scientific evidence, is the fundamental concept behind evidence-based restoration. After data collection and evidence summarization of the object to be restored in the second phase, the restoration team uses critical thinking to evaluate the source level, authenticity, significance, relevance, and other aspects of the collected evidence. This is done to ensure that the evidence is of high quality and relevant to the research problem to make the "best decision" for virtual restoration.

A four-dimensional matrix is created in the virtual restoration's evidence assessment section, and its basic components include the restoration hierarchy of heritage in the X dimension (hierarchical grouping of evidence types and the pyramidal hierarchy to which they belong), the quantity of evidence in the Y dimension, the completeness of evidence in the Z dimension, and the expert judgment of evidence in the C dimension. Using the information composite and synergy of the multidimensional matrix to achieve the reliability of restoration evidence in a comprehensive assessment, the reliability index of virtual restoration evidence is set forth as in Eq. (1). This dimension, U, makes up a multidimensional spatial judgment system for architectural restoration evidence assessment (1).

1 $$E=\sum \frac{\left(L+G\right)}{2}*Q*I*e$$

E, Reliability index of virtual restoration evidence (Evidence). L, Hierarchy of evidence for building restoration (Level). G, the pyramidal grade (Grade) to which the evidence of architectural restoration belongs. Q, the quantity of evidence (Quantity) for building restoration. I, Evidentiary integrity of architectural restoration (Integrity). e, The index of expert judgments of the evidence (expert).

**Fig. 10:**
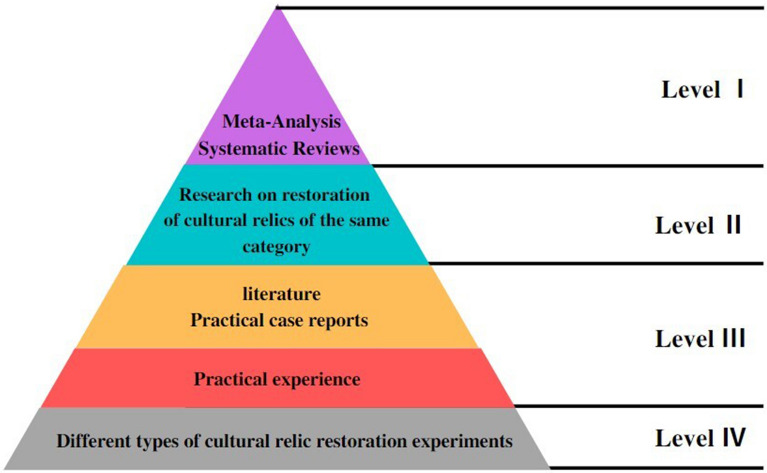
Pyramidal grade

Because the evidence comes from multiple sources, it cannot be evaluated in a single hierarchical manner at the source. As a result, the "evidence pyramid" was developed, and the average of the two was used as the hierarchical X dimension of the repair evidence. This study proposes the modification of the "evidence pyramid," which was first introduced by SUNY Downstate Medical Center (SUNY Downstate Medical Center, USA) in 2001, to assess evidence in "evidence-based medicine." Based on this, this study suggests an "evidence pyramid" that is modified for "evidence-based repair" and incorporated into the reliability index of evidence, as shown in Fig. 10.

Today, the precision of machines allows for the accurate assessment of evidence but not flexibility, which is why an index that requires human judgment—expert assessment of evidence—in the four-dimensional U dimension matrix of evidence assessment was introduced. Architectural heritage has hidden sentimental value.

#### Virtual restoration guidance practice

A crucial part of "evidence-based restoration" is "virtual restoration," which simulates the restoration of a building's past using evaluated data to determine whether and to what extent the objectives of the first phase can be accomplished. If the results support restoration goals, then the restoration moves on to the second phase of data-based research, where the evidence is adjusted and analyzed until the virtual restoration goals are met. If the computerized response to the research questions is accurate and the goals are met, then the virtual restoration plan is valid, and the restoration moves to physical restoration.

The two types of virtual restoration are ornamental restoration and structural repair. The outsides of historically significant buildings are frequently restored using semantic automated restoration based on deep learning, while external virtual restoration is frequently handled using image processing technology. Wang et al. [54] performed the virtual restoration of cracked murals using top-hat to extract crack information, queer segmentation, morphological operations, and sample-based restoration algorithms, while Zou et al. [55] used U-Net to convert the unrecognizable color restoration problem into a semantic segmentation problem for some old and dilapidated buildings, where the naked eye could no longer distinguish colors.

It is crucial to restore the structural integrity of historic buildings. If structural heritage is not properly restored in terms of appearance, then it will lose its original flavor and value and look inappropriate, while improper structural restoration will cause such heritage to vanish. Because wood structural damage mechanisms are more complex, it is reasonable to integrate multidisciplinary, clear repair ground research problems, the development of a correct and reasonable repair strategy, technical solutions and engineering systems, and virtue engineering. Cracks, bending, rolling, tenon pulling, and other forms of structural damage are common in wooden structures; traditional structural repair and reinforcement rely on the craftsperson's experience and judgment [56–58]. The procedure and method for the virtual structural restoration of historic buildings can be summarized as the integration of analytical data through data-based research, combined with infrared thermography, three-dimensional stress waves, and other technologies, further combined with related experimental investigations, surveying the structure or component damage characteristics information, and the construction of a three-dimensional refinement of the building information model [59, 60]. Finite element static analysis and virtual repair are used to evaluate the safety performance before and after repair. Additionally, various cumulative damage time-varying models have been built based on member damage information to assess the reliability of member repair and provide reliable theoretical research for physical repair design [61, 62]. This research offers a trustworthy theoretical foundation for physical repair design.

#### Post feedback

The notion of "post repair feedback," which is taken from the idea of "feedback" in control theory, is proposed in this work based on the use of evidence-based design theory in the virtual restoration of architectural heritage. Post occupancy evaluation (POE) is the process of gathering information on a building's spatial performance, physical performance, and user perceptions after it has been completed to provide designers, managers, and users with suggestions and better architectural standards [41, 63]. Feedback after restoration is used to assess the accomplishments of both the virtual and physical restoration of architectural heritage, to verify the veracity of the "evidence," and to enhance the "evidence" to strengthen its validity and trustworthiness.

The evaluation of the restoration impact of the heritage body and the discovery and restoration of the additional social and cultural values of the heritage are the two primary divisions of post restoration feedback material. The core component is the restoration of the heritage ontology, and as shown in Table 3, the evaluation criteria for each stage in the following evaluation of social benefits and technical indicators are established based on the values of the restoration of the heritage body [63]. Please refer to the table for more information.

**Table 3:** Core components of the restoration of the heritage ontology

Evaluation dimension	Category of evaluation content	Content description
Evaluation of body treatment effect	Effect of protective measures	Effectiveness of conservation measures against heritage disease and aging and whether they pose additional risks
	Coordination with the authenticity and integrity of the heritage	Whether conservation or reuse measures have a negative impact on the heritage's historical, artistic, technological, etc., values
Social benefit evaluation	Expression of value such as heritage, history and culture	Whether to strengthen the cultural symbolism of the heritage or strengthen the emotional relationship between the masses and heritage, cultural cognition, etc
Evaluation of technical indicators	Structural and constructive state	Determination and simulation calculation of structural stability and safety of heritage ontology, etc
	Operating energy consumption	Dissipation of water, electricity and materials in operation after renovation and whether green energy-saving measures can be taken, etc
	Disaster prevention measures	(Especially architectural heritage) whether basic fire protection needs are met, whether there is a natural disaster risk such as address, flood, etc., and whether it can be dealt with
Protection system	Protection system	Whether the management system in the process of heritage protection and operation is reasonable
	Management mode	Whether the current operation mode of the heritage meets the protection needs and maximizes the value added of the heritage, etc

The evaluation of the effect of the restoration of the heritage body is a technical field, and the long-term monitoring of the heritage body can be monitored in real time by means of the placement of sensors [64]. The methods of nondestructive testing are photogrammetry, 3D laser scanning, ultrasonic testing and other methods [65], ultrasonic detection [66] ground-penetrating radar [67]. Moreover, point cloud [68] seminondestructive testing requires a minimally invasive examination of heritage, such as resistance drilling techniques [69]. Laboratory testing is generally a microscopic experimental analysis of the material part of heritage objects [70]. The evaluation and feedback on the additional social and cultural values of heritage should be based on a combination of qualitative and quantitative paradigms, introducing modern science and technology and the concept of big data, using eye-tracking devices to collect data and evaluate user preferences and perceptions [71]. The evaluation and feedback of the social and cultural impact can be made by combining expert evaluation and hierarchical analysis and by capturing open data from the internet [41, 72]. The project also combines expert evaluation and hierarchical analysis to capture open data from the web to evaluate and provide feedback on the social and cultural impact.

The restoration process of heritage should be integrated as a directed case study, involving research and conservation, and become a rigorous evidence-based system containing high-frequency feedback itself [73]. The results of the restoration process should be recorded and integrated into an evidence system, combined with a registration system, to build a database of evidence-based evidence of heritage restoration [74]. It should a system of evidence that is used to record and integrate the post restoration feedback results, which is combined with a registration system to build a database of evidence-based heritage restoration evidence and is used as special evidence for the evidence-based practice of conservation and reuse in each case, to enter the next case cycle.

## Results

### Case introduction

The Bagong House in Wuhan, Hubei Province, China, which has rich historical value and was named one of the city's first batches of outstanding historical structures in 1993, was added to the "Preparatory List of China's World Cultural Heritage" by the State Administration of Cultural Heritage in 2019 and was added to the eighth batch of Hubei Province in 2021, serving as the case study. The Bagong House is separated into two sections called Da Bagong and Xiao Bagong by J. K. Barnoff. J. K. Barnoff and Zino Barnoff each contributed to its construction. It is an example of a high-class apartment building in Wuhan's Hankow tenement district and shows the development of Wuhan's modern architectural style as well as the city's early real estate development and business strategy. Table 4 displays the architectural layout of the Bagong House.

**Table 4:** Overview of the Bagong House architecture

Overview of the Bagong House architecture	
Construction time	The "Big Bagong House" was built in 1910, but it is unknown when the "Little Bagong House" was built
Designers	Jingming Foreign Affairs
Constructors	Yongmaochang and Kuanchang Construction Factory
History functions	High-rise apartment/bank staff dormitory/ground floor commercial/residential building
Structure form	Brick and concrete
Roof form	Sloping roof (Big Bagong), flat roof (Little Bagong)
Number of floors	One underground, three above ground, with a partial addition to the fourth floor
Land area	Approx. 2500 m^2^
Building area	Approx. 10,000 m^2^

The Bagong House was constructed many years ago, and although it has been used for almost a century, it has undergone numerous expansions and transformations to become the building it is today. The roof of the house, as well as a few structural situations, the building layout, along with the street facade, and the original design differ from one another. The building has several structural safety issues, such as structural aging and corrosion, and there are safety dangers according to a housing inspection report. The conservation and repair project of the Bagong House is urgent and necessary due to the local alterations of the building's function, layout, and structure, the original equipment department's failure to meet the building's normal use requirements, and the damage to the building's key conservation parts and conservation projects. Part II of this study's "Evidence-based Design in Virtual Restoration of Architectural Heritage" section uses the restoration of the Bagong House as an example.

### Empirical studies

#### Clearly defined objectives

**Fig. 11:**
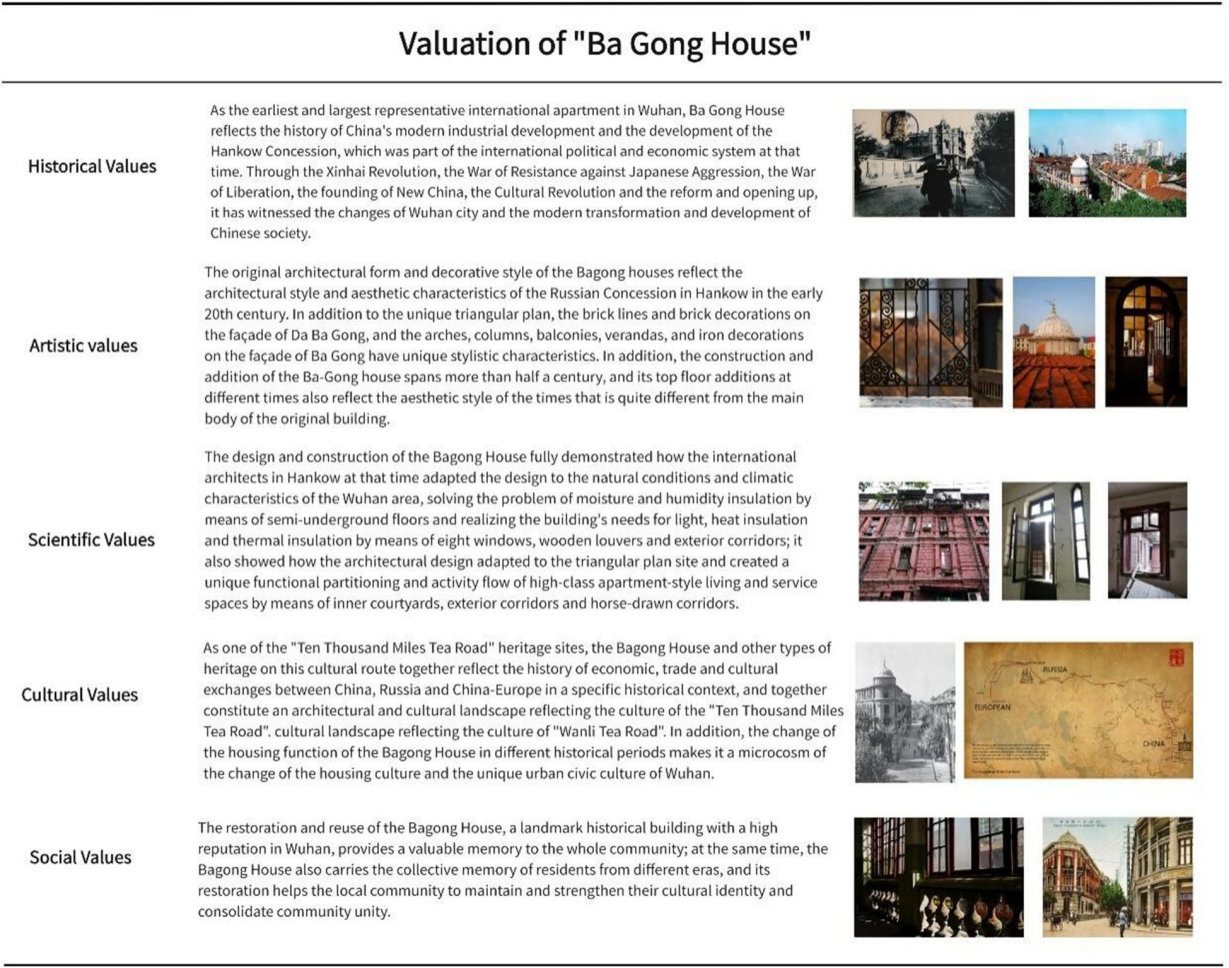
Valuation of the Bagong House

Heritage value assessment and restoration objectives are the two main sections of the "Defining Objectives" section. In accordance with the Guidance on Heritage Impact Assessments for Cultural World Heritage Properties published by UNESCO and ICOMOS, the historical value, artistic value, scientific value, cultural value, and social value of the Bagong House were evaluated, the results of which are displayed in Fig. 11.

The restoration team established the broad restoration goals as follows, using the heritage value assessment findings and the Bagong House's present state of use:

1. To recreate its overall historical aspect by preserving and restoring its classically inspired exterior and interior spaces as well as its distinctive embellishments.
2. To ensure structural security through preservation and repair for future use over a longer period of time.
3. The revitalization of cultural heritage buildings will be accomplished through a variety of newly implemented functions, and while honoring and reproducing the spirit of the place, a cultural tourism destination of international level and influence will be created in Wuhan, igniting "new life in the old city".

#### Evidence-based research

**Fig. 12:**
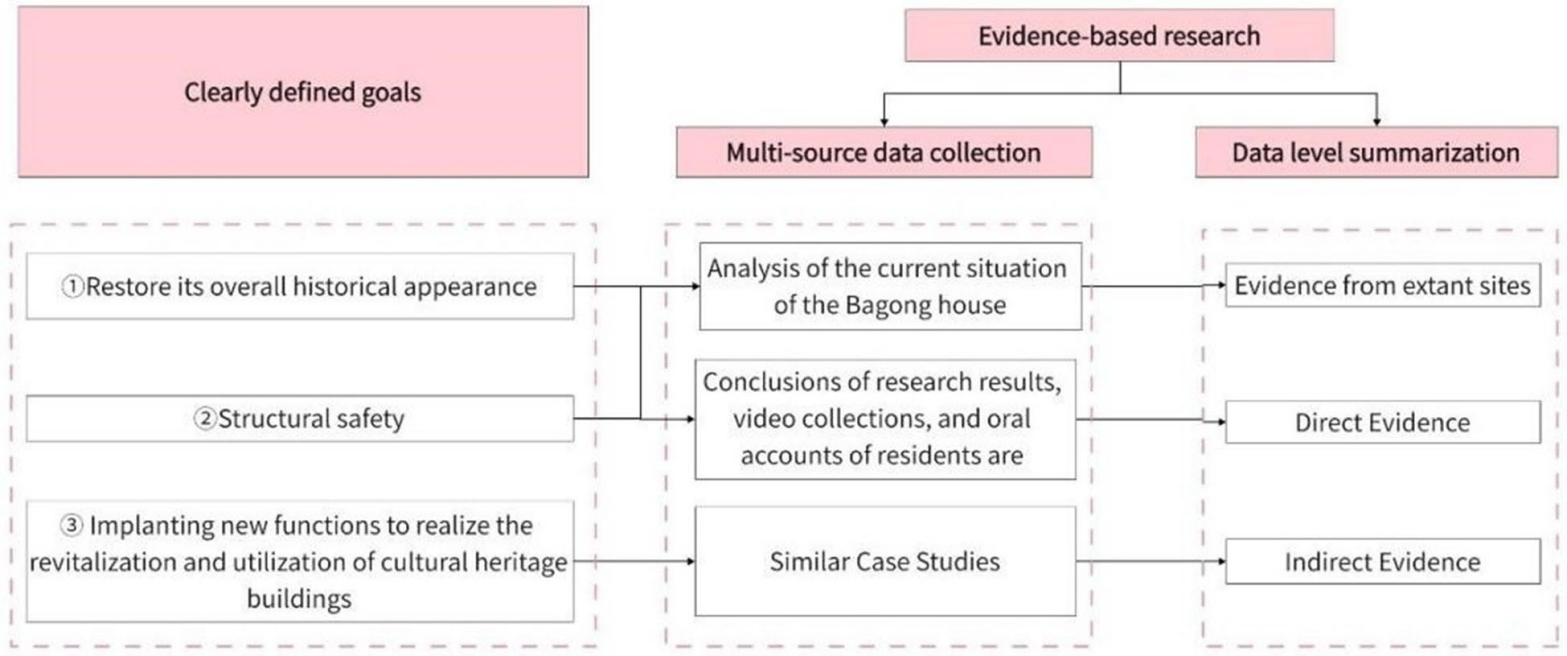
Evidence correspondence

Evidence was gathered from existing sites in the section on evidence collection, including direct and indirect evidence, and Fig. 12 illustrates how these two types of evidence correspond.

1. Existing site evidence. This section is analyzed primarily by drawing information from the Bagong House's current state, which is a mapping of the site's actual state and reflects its objective authenticity. To ensure that the photos taken can meet the needs of 3D reconstruction, information is acquired primarily through the use of airborne camera imaging systems to gather external and environmental impact data. The relevant current state information is shown in the Fig. 13 by using the camera to collect pieces of local information one at a time, which are then used to analyze the local status of the building interior.

**Fig. 13:**
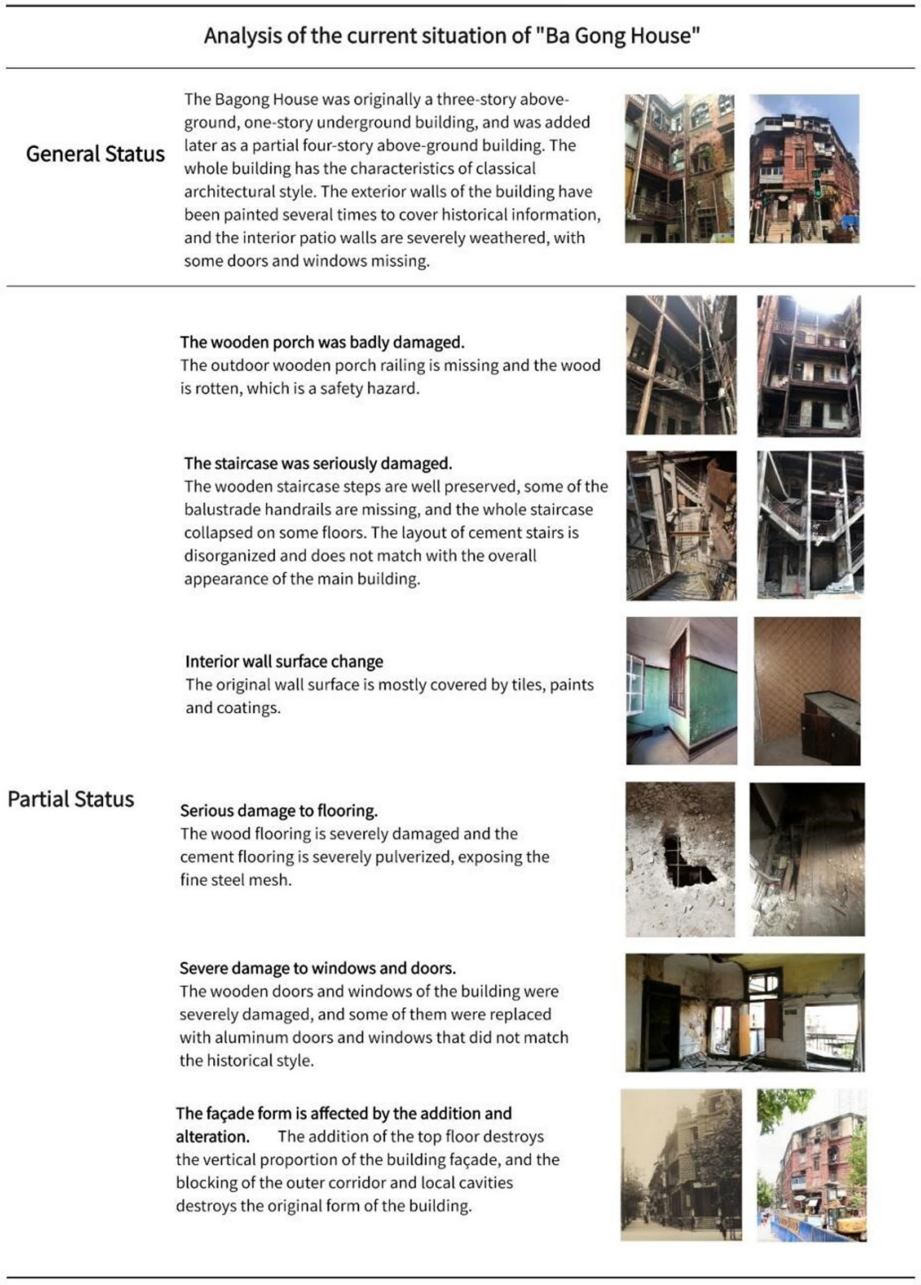
Analysis of the current situation of the Bagong House
2. Direct evidence. To prepare for the clarification of 1 in the target, a portion of evidence collection and collation is shown in Fig. 14. This part focuses primarily on speculating on the original layout and appearance of the Bagong House through pertinent image sets and oral recollections of nearby residents from its construction to the present.

**Fig. 14:**
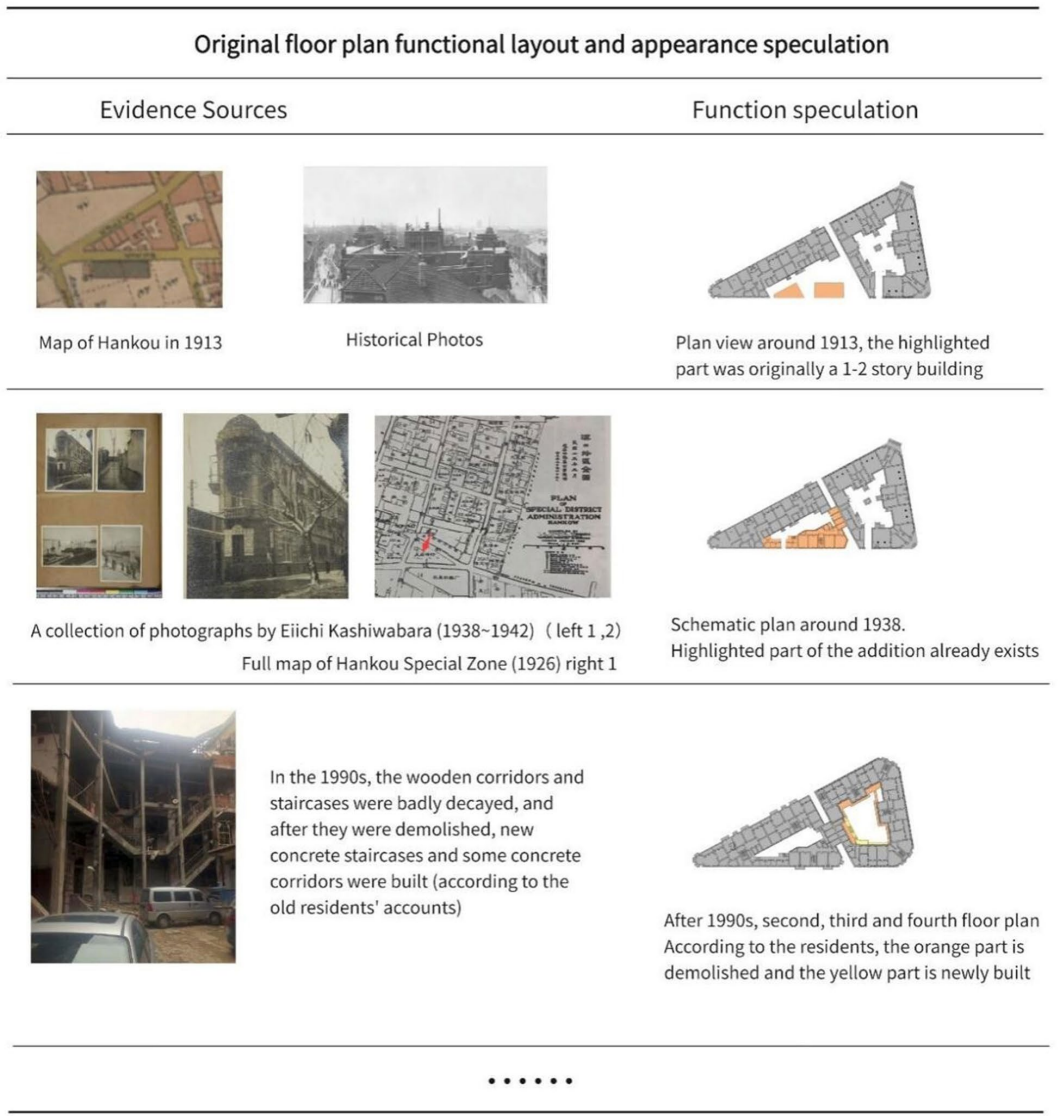
Original floor plan, functional layout and appearance speculation
3. Circumstantial evidence. Indirect evidence from cases such as the Bagong House, the nearby Holy Church Bookstore, and the out-of-town Wukang Building (formerly Normandy Apartments) is used primarily to support the third restoration objective, the adaptive use of cultural heritage buildings through newly implanted multiple functions. Before the Bagong House, both of the above sites had been restored. It is possible to instill new functions in cultural heritage buildings that can preserve the status quo of the heritage site and have a positive impact on its adaptive use, as shown in the Fig. 15, by conducting a comparative analysis of similar buildings.

**Fig. 15:**
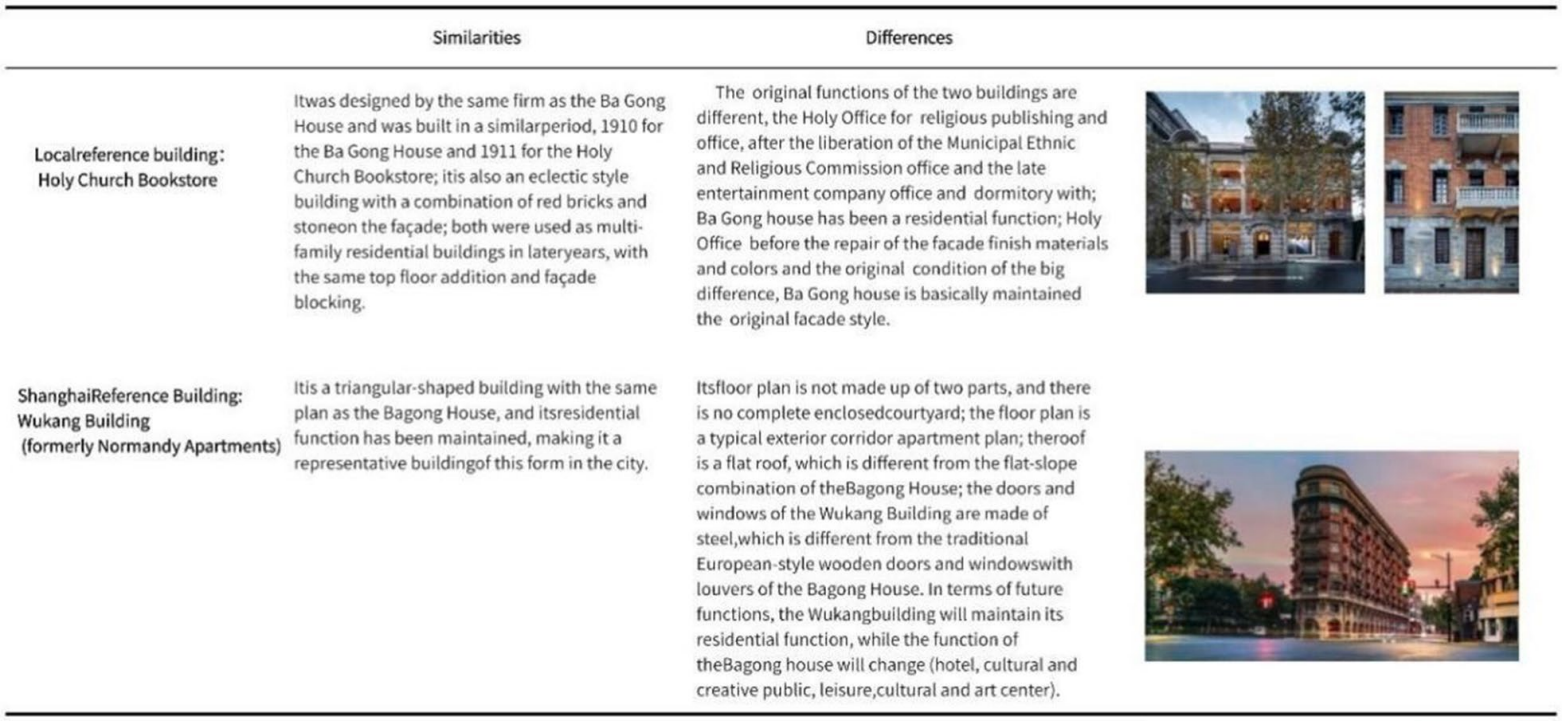
Case study (Sacred Church Bookstore (Photo credit: https://www.credaward.com/project/179276/) Wukang Building (Photo credit: https://www.thepaper.cn/newsDetail_forward_11637646).

#### Evidence assessment

**Fig. 16:**
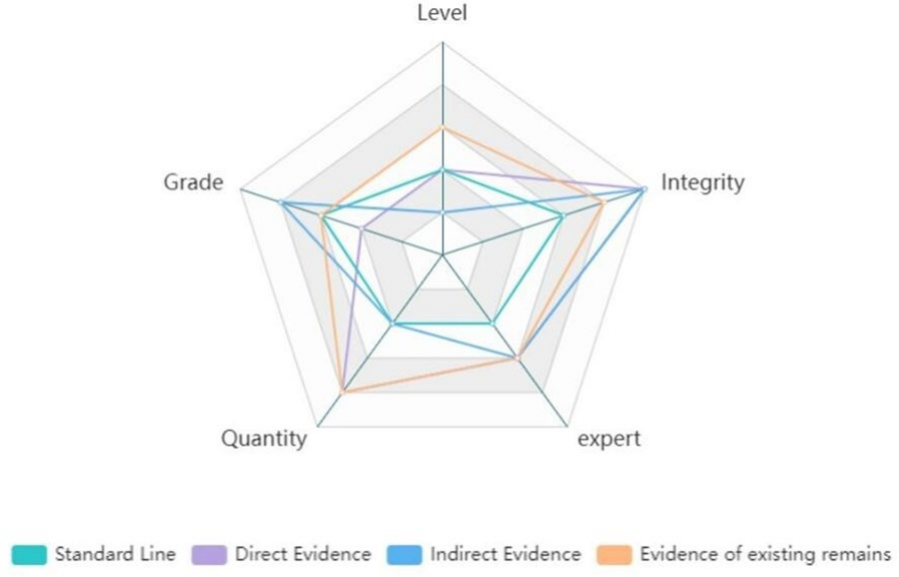
Evidence assessment

This section assesses and analyzes the evidence gathered in the "Evidence-based research" section and presents the findings as a radar chart in conjunction with the "Evidence assessment" section, as shown in the Fig. 16 The level, grade, quantity, integrity, and expert described in the "Evidence assessment" section are all indicated on the standard line, which is orange in color. Additionally, it should be noted that each case involving architectural heritage has characteristics, and the evaluation of the evidence in the assessment process can change depending on how the decision is viewed. For these reasons, researchers should take a comprehensive approach to the evaluation of such evidence.

#### Virtual restoration guidance practice

**Fig. 17:**
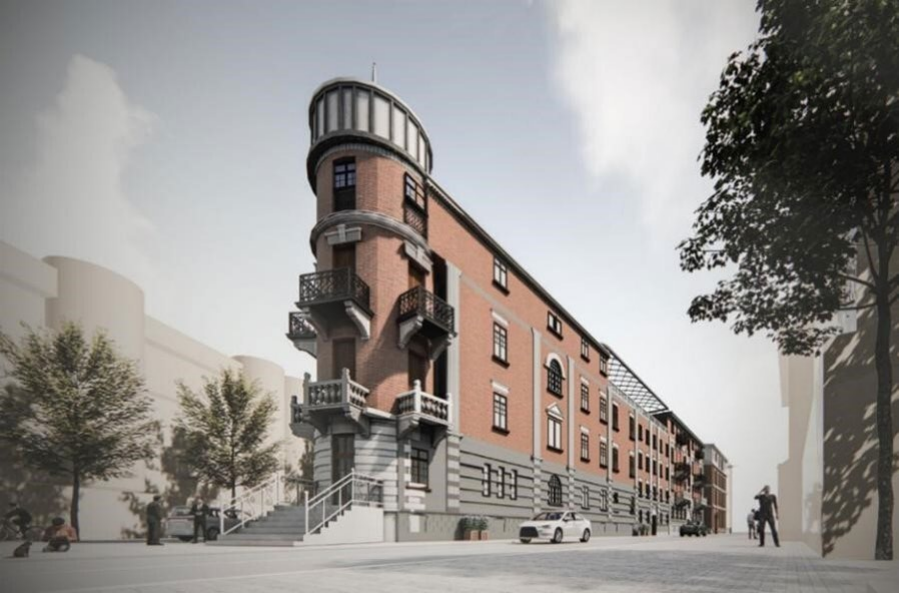
Virtually restored Bagong House

The Bagong House's restoration phase mostly entails the installation of additional functions, structural reinforcement, and general external and interior design, according to the restoration's goals. As a result, the objective in the virtual restoration guiding practice phase is to determine the damaged exterior and interior decoration and structure through evidence-based research and evidence assessment, to design the implementation of new functionalities, and to then guide the practice. Figure 17 depicts the Bagong House's overall virtual renovation.

**Fig. 18:**
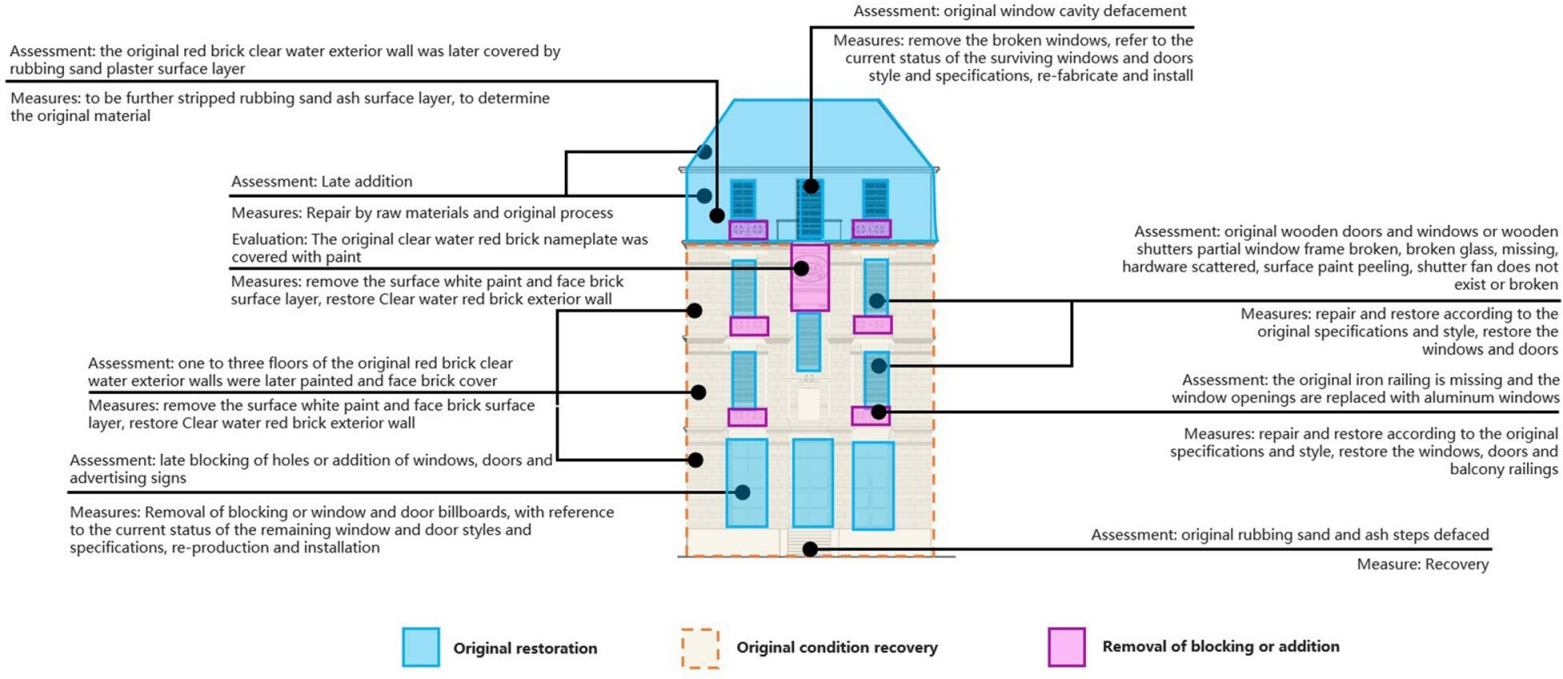
Virtual restoration and restoration guide diagram

**Fig. 19:**
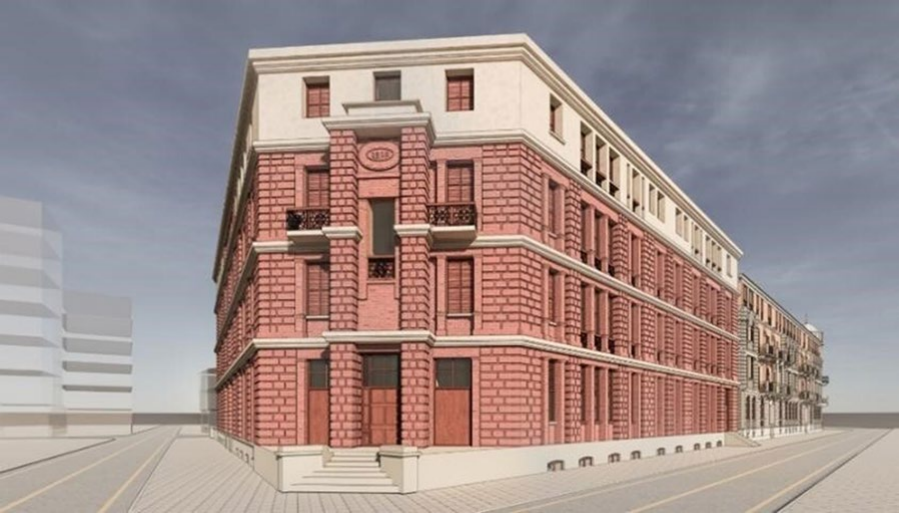
Virtually restored Bagong House elevation

Extrapolation based on exterior damage. This study uses the southwest façade of the Bagong House as an example to explain the repair plan and restoration guidelines for the façade. These plans are based on the existing site evidence and direct evidence. The former involves a comprehensive examination of the façade's design and construction. On the southwest façade of the Bagong House, there is a red brick façade on the exterior walls of the first to third floors, rubbed sand plaster façade on the exterior walls of the later addition of the lower four-story building with two low sides, and there is a late addition. The restoration goals are to restore the rubbed sand plaster footings, restore the red brick façade on the exterior walls of the first to third floors, and repair the rubbed sand plaster façade on the exterior walls. Figure 18 displays a virtual restoration and restoration guide diagram, Fig. 19 is after virtual restoration.

**Fig. 20:**
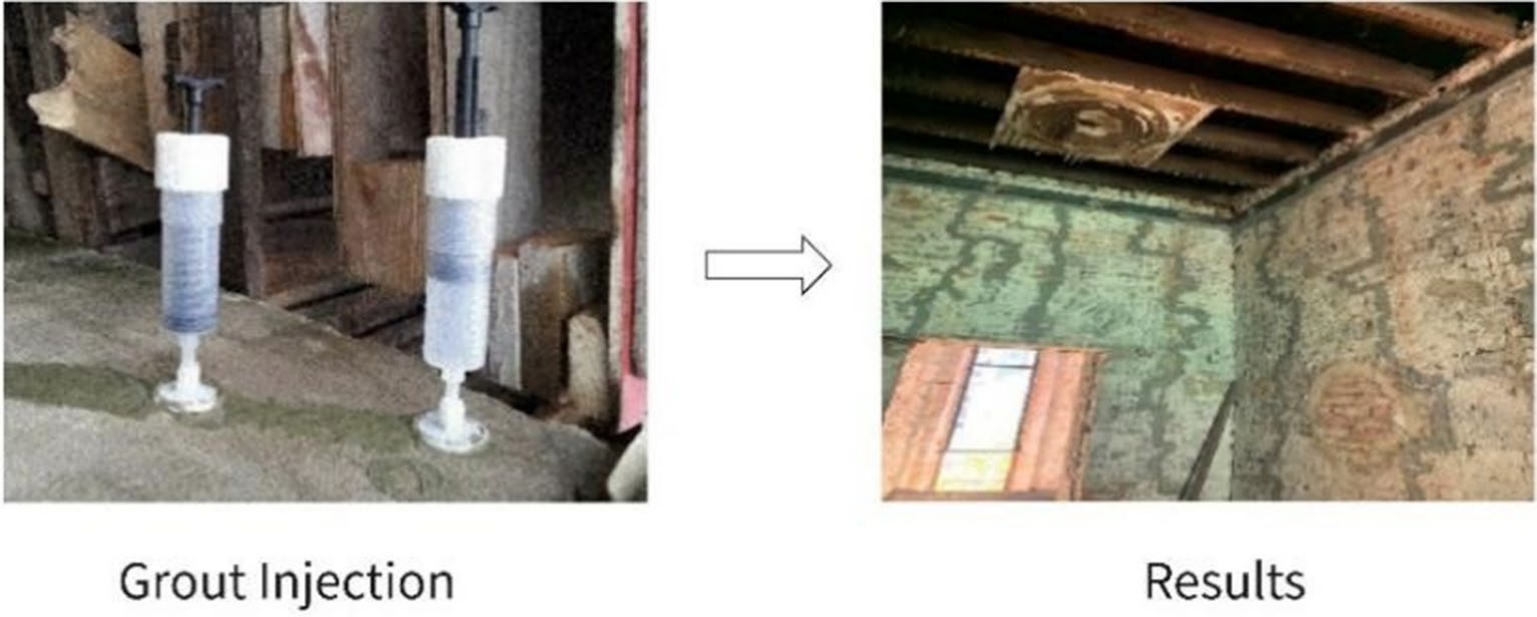
On-site restoration techniques

The structural part of the Bagong House adopts mainly structural reinforcement for protection. Taking crack repair in concrete engineering as an example, the main process is to clean up the crack → install grouting nozzle → seal the crack → conduct a pressure gas leak test → apply grouting pressure → apply sealing treatment. The effect is shown in the Fig. 20 below.

**Fig. 21:**
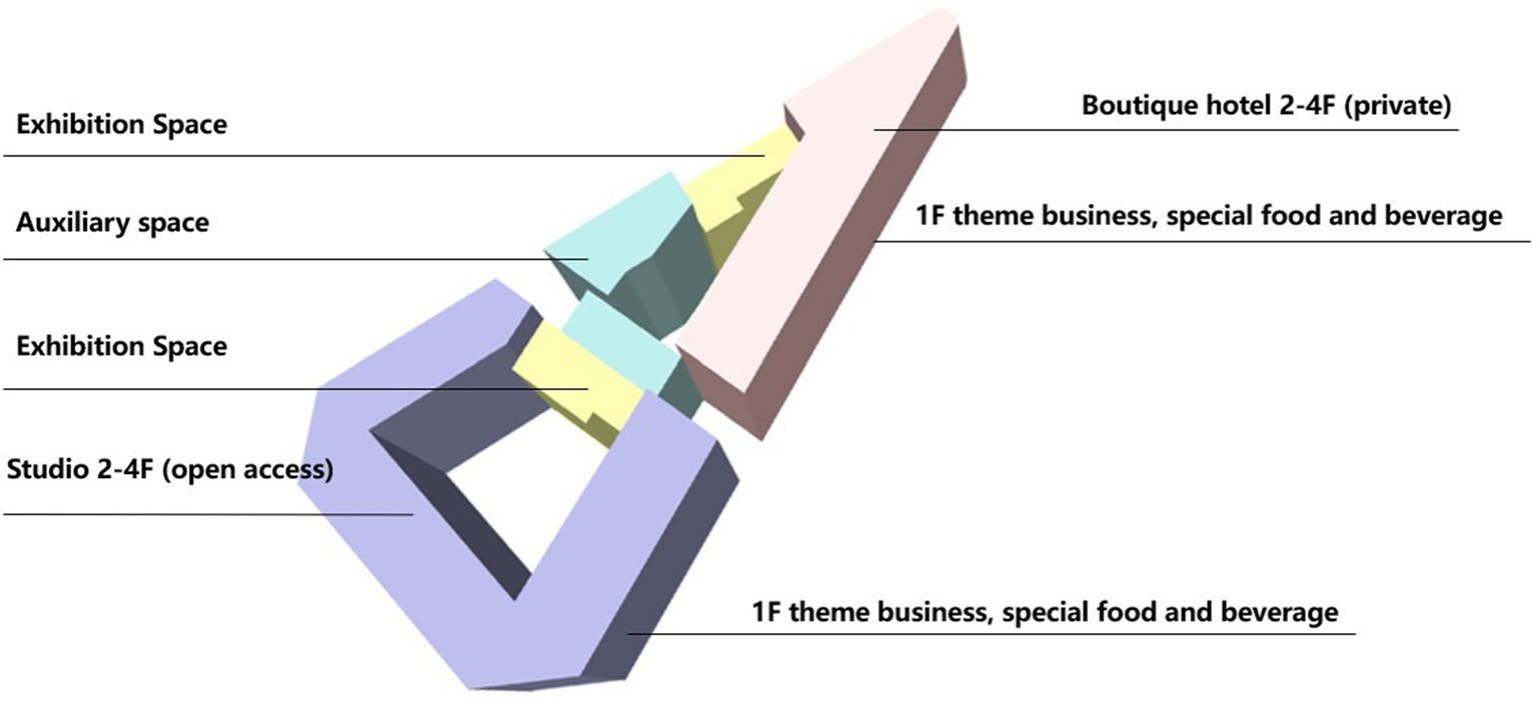
Partition mode

**Fig. 22:**
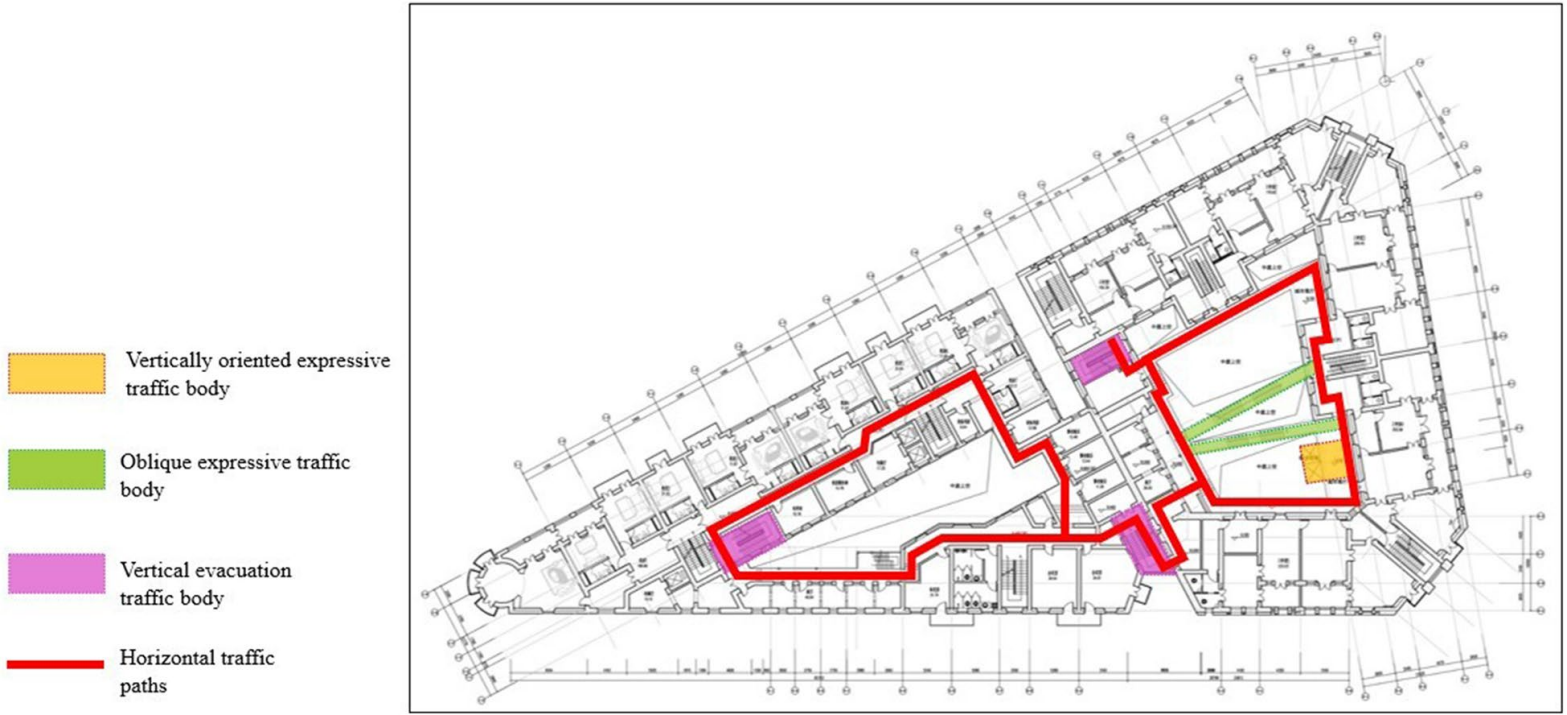
Route map

Restoration experts have defined the main business mode of the Bagong House as an ecological circle integrating a cultural and creative gathering place, cultural and leisure services, and microtourism, the zoning model of which is shown in the Fig. 21 below. This was done through the study of the Sacred Church Bookstore and the Wukang Building as indirect evidence. The restoration idea for the Bagong House is explained using traffic flow as an example. To meet the requirements for a fire evacuation, a newly added vertical evacuation stairway is coupled with an external horizontal traffic corridor to form a horizontal circular path parallel to the interior walkway of the building, thus restoring the original historical scene, as shown in the Fig. 22. In addition, the horizontal path is creatively coupled to the expressive diagonal and vertical tour traffic bodies, producing a highly diverse tour path and a very intriguing, three-dimensional urban public living room.

#### Post feedback

The ontology of the Bagong House was recently restored and has not yet been formally opened to the outside world, so many spatial performance and social benefit evaluations cannot be fully collected. The overall effect on post feedback requires a certain amount of time for the ontology to operate, as short as one and a half years or even several years. Therefore, only the issues that require attention in the post restoration feedback of architectural heritage, as described in “Post feedback” section, are suggested in the post feedback section of this paper.

## Discussion and conclusions

This article investigates the theoretical and practical paths of evidence-based design in the virtual restoration of architectural heritage, combining closed-loop theory to propose the alternation of evidence creation and evidence use procedures to ensure the success of the virtual restoration of architectural heritage. The use of evidence-based design in the virtual restoration of architectural heritage is being investigated. A scientific, humanistic, and workable theoretical foundation for the restoration of architectural heritage is provided by the combination of evidence-based design and virtual restoration. Such work also offers new ideas that can be used as references for the restoration of other cultural heritage sites, which has significant application value and practical significance.Authors’ contributions: Journal standard instruction requires the statement "All authors read and approved the final manuscript." in the “Authors’ contributions” section. This was inserted at the end of the paragraph of the said section. Please check if appropriate.Thank you very much for the reminder and have added "All authors read and approved the final manuscript." to the "Authors' contributions" section.

Although this topic has been examined in the context of empirical cases, this study constructs a theoretical exploration of the practical route of evidence-based design in the virtual restoration of architectural heritage in the context of the information age. Several theoretical issues derived from the following need to be further studied.

1. Transparency of evidence in evidence-based research. However, any approach to preserving and restoring architectural legacy must adhere to the concept of authenticity, and the virtual restoration of architectural history is fraught with uncertainty. The authors of this study suggest virtual restoration based on evidence-based theory, which is essentially a continuous human decision-making process and inherently introduces some degree of unreliability and uncertainty [75]. The decision-making process should be as formulaic and open book as possible to reduce uncertainty and increase the overall credibility of virtual restoration.
2. New developments in the evidence and outcomes of architectural heritage restoration are not static, and the majority of the evidence data gathered in the evidence-based research component is of an immediate nature because it is derived from the study of particular issues and occurrences over a predetermined period of time. However, the applicability and quality of the evidence may vary when new concerns and the external decision-making environment develop. The evidence database needs to be updated on a regular basis to guarantee the variety of kinds of evidence and the relevance of the evidence content. The greatest benefit of the closed-loop theory introduced in this study is that it allows new evidence to emerge, and post feedback is an important method of evidence updating. As most architectural heritage has a history of thousands of years, the process of evidence-based research is inevitably challenged by a large amount of missing evidence. Only by regularly updating the evidence can scientific practice be led, and updating the evidence is a key factor in encouraging evidence-based design in the restoration of architectural heritage.
3. The relationship between technology and culture. According to Martin Heidegger, "Technology is not by its nature technology" [76]. Technology has unforeseen implications and risks, which warrant consideration and action. The emphasis on obvious risks has, however, somewhat obscured the underlying threats of cultural and moral degradation [20]. Technological advances have caused unheard-of changes in how cultural heritage is preserved and restored, but their use should not alter conservation's fundamental principles; rather, they should strengthen such principles. The preservation and restoration of architectural legacies is primarily a cultural issue rather than a technological one, and in the evolving digital age, the word "culture" has taken on a new, larger meaning that goes beyond heritage itself. In this period, we also need to think carefully about how to employ digital technology to protect and restore architectural legacies in a rapidly changing culture.

## Data Availability

The datasets generated and/or analyzed during the current study are available from the corresponding author on reasonable request.
